# Weibull Modeling of Controlled Drug Release from Ag-PMA Nanosystems

**DOI:** 10.3390/polym13172897

**Published:** 2021-08-27

**Authors:** Carmelo Corsaro, Giulia Neri, Angela Maria Mezzasalma, Enza Fazio

**Affiliations:** 1 Department of Mathematical and Computational Sciences, Physics Science and Earth Science, University of Messina, Viale F. Stagno D’Alcontres 31, I-98166 Messina, Italy; ccorsaro@unime.it (C.C.); angelamaria.mezzasalma@unime.it (A.M.M.); 2 Department of Chemical, Biological, Pharmaceutical and Environmental Sciences, University of Messina, Viale F. Stagno D’Alcontres 31, I-98166 Messina, Italy; giulia.neri@unime.it

**Keywords:** Ag nanoparticles, Poly(methacrylic acid) sodium salt, photoreduction process, Weibull function, statistical distributions, drug nanocarrier, smart nanodevice

## Abstract

Traditional pharmacotherapy suffers from multiple drawbacks that hamper patient treatment such as antibiotic resistances or low drug selectivity and toxicity during systemic applications. Some functional hybrid nanomaterials are designed to handle the drug release process under remote-control. More attention has recently been paid to synthetic polyelectrolytes for their intrinsic properties which allow them to rearrange into compact structures, ideal to be used as drug carriers or probes influencing biochemical processes. The presence of Ag nanoparticles (NPs) in the Poly methyl acrylate (PMA) matrix leads to an enhancement of drug release efficiency, even using a low-power laser whose wavelength is far from the Ag Surface Plasmon Resonance (SPR) peak. Further, compared to the colloids, the nanofiber-based drug delivery system has shown shorter response time and more precise control over the release rate. The efficiency and timing of involved drug release mechanisms has been estimated by the Weibull distribution function, whose parameters indicate that the release mechanism of nanofibers obeys Fick’s first law while a non-Fickian character controlled by diffusion and relaxation of polymer chains occurs in the colloidal phase.

## 1. Introduction

Nowadays several efforts are focusing on the development of eco-friendly approaches to reduce the environmental impact of industrial production. To reach this goal, bottom-up techniques concerning the design of nanodevices able to efficiently perform one or more tasks [1] in the field of artificial photosynthesis [2,3], biomedicine [4,5], non-invasive diagnostic techniques [6,7], food packaging [8,9] are receiving ever-growing attention due to the versatility in the modulation of their properties. On the other hand, the use of biomaterials is crucial to preserve human and the ecosystem’s health. However, the low chemical-physical stability, short lifetime and poor mechanical properties of these materials limit their extensive employment. To overcome these hurdles, the incorporation of materials of different nature in a bio matrix allows achievement of hybrid nano-systems showing improved properties compared to the starting ones [10,11]. In the biomedical field, the necessity to provide smart drug nanocarriers able to keep the drug concentration within its therapeutic window avoiding under-dosing (inefficient treatment) or over-dosing (cytotoxicity) phenomena such as multidrug resistance effects, led to design stimuli-responsive drug delivery systems [12,13,14].

Among the several kinds of nanodevices, polymer matrix based nanocomposites have emerged as the most promising and viable systems in the food industry [15] as well as in drug nano-delivery systems [16]. The incorporation of pharmaceuticals into electrospun polymeric fibers has several advantages such as high porosity and high surface area to volume ratio, obtaining a system similar in structure to extra cellular membrane [17]. Further, to increase drug collection/release efficiency in a nanofiber system, controlled wetting properties are desirable [18]. Thus, fiber systems are employed to reach both immediate and controlled drug release processes [19,20].

At the same time, stimuli-responsive bio-based polymeric systems are gaining considerable attention as intelligent versatile tools that show great potential in the food packaging field. The idea is that when a beneficial interaction between the packaging, environment and food occurs, the bioactive compound is released under control [21]. Moreover, the development of multifunctional polymer-based nanocomposites exhibiting several functionalities combined in a single device is one of the most favorable approaches to reach new smart materials [22,23,24]. In this perspective, silver nanoparticles (Ag NPs) are considered one of the most promising candidates in biomedical applications thanks to their multiple functions [25,26]. In addition, recent research has shown promising results for their innovative and potential applications in the food field, which also include their integration into intelligent food packaging systems and their direct incorporation into food matrices as a flavor carrier system [27]. However, despite the remarkable advantages and innumerable positive properties of Ag NPs, safety concerns have been raised over their usage because they pose potential environmental hazards [28]. It is worth mentioning that Ag NPs can affect aquatic photosynthetic organisms [29], and they are also able to induce oxidative and genotoxic damages on organisms and cells [30]. Therefore, the prevention of Ag NPs diffusion in the biosphere is a pivotal step in the design of new and effective systems based on Ag NPs.

To achieve this goal, particular attention has been devoted towards the use of polyelectrolytes which act both as reducing and stabilizing agents, avoiding the use of surfactants. Synthetic polyanions are widely employed in the medical field as arthritis adjuvants, promoters of the endosomal escape, modulators of the phagocytic activity and drug delivery systems [31,32]. Among these synthetic polyanions, poly methyl acrylate (PMA) offers several advantages such as: (i) water solubility, (ii) capping agent thanks to the ability to wrap of metal clusters [33] and (iii) good biocompatibility, which is proved by its employment in biomedicine and biotechnology fields [34,35,36]. Moreover, it was demonstrated that the stability of its compact form is the result of a combination of several forces including (i) short-range van der Waals forces correlated to methyl side chains, hydrophobic interactions and (ii) hydrogen bonding, which involves the carboxyl functional groups [32]. Lastly, PMA is characterized by a water contact angle of ~73° [37], a higher value compared with that of PEG (~39° [38]) and poly(methyl methacrylate) (PMMA), (~68° [39]). Therefore, its low hydrophilicity allows maintaining its mechanical properties when absorbing water. Considering the relevance of wettability properties in the drug release process, the intrinsic physical and chemical features of PMA make it a promising candidate as a drug nanocarrier.

Herein, Ag-PMA colloidal solution was prepared by a green and eco-friendly UV photoreduction procedure [40,41]. PMA acts as capping agent allowing well-defined and low distribution in the size of the hybrid nanocolloid (~10 nm diameter). Then, Sorafenib Tosylate (SFT), a promising hydrophobic (log P = 3.8) anticancer drug [42], was embedded in Ag-PMA matrix. The drug release profiles of the prepared nanocolloid and the corresponding electrospun Ag-PMA nanofibers scaffold, were analyzed by a Weibull statistical distribution function, gaining physical insights on the mechanisms governing drug release. Since the original work of Waloddi Weibull [43], the Weibull distribution has been applied to a great number of mechanisms for studying the kinetics of heterogeneous processes [44], also demonstrating its overall superiority for fitting the drug release by nanosystems [45]. The model-independent statistical simulation of in vitro release profiles was introduced by Koester et al. [46], who also established the supremacy of the Weibull model over other competing statistical models through linear regression analysis [47]. Generally, logistic approaches achieve a high degree of accuracy, although they require several parameters and a certain degree of complexity during the fitting process. It is worth to mention that, different mathematical models are usually used as tools to predict the drug release kinetics, investigating their applicability in specific time intervals [48]. In this work, after having checked some mechanistic models including power law and Higuchi models, we have chosen to use the continuous Weibull function (simpler than the logistic function) which fits the whole release process, providing a statistical description of it. The Weibull function has shown, in fact, better fitting performances with a good degree of accuracy and, above all, it allowed us to find some correlations among its parameters, the release process features and, in turn, with the morphological characteristics of the studied systems. The basic idea is to statistically interpret the normalized curve for the drug release kinetics as a cumulative distribution function (CDF), since its trend is monotonic increasing and reaches 1 (or M_∞_, the maximum released drug amount) at large times. In such a way, we interpret the drug release kinetics as the probability of the drug to be released at a time t. Indeed, we use the Weibull cumulative distribution function (which is the complementary function of the stretched exponential form [49]), to obtain information about the average relaxation time and the Weibull shape factor, strictly related to the specific release mechanism. Furthermore, the time derivative of the Weibull CDF, representing the Weibull Probability Density Function (PDF), provides the probability of reaching the maximum of drug release at a time t (corresponding to the drug release speed). Then, a correlation among Weibull parameters, release mechanisms and samples properties has been carried out. All that is essential for a better understanding of the release mechanisms and serves as a basis for designing innovative PMA based controlled release type formulations.

## 2. Materials and Methods

Poly(methacrylic acid) sodium salt ([CH_2_C(CH_3_)(CO_2_Na)]n, Mw = 9500) and silver nitrate powder (AgNO_3_), were purchased from Merck (Milan, Italy) and used without further purification. Sorafenib Tosylate (SFT) was offered by the Department of Chemical, Biological, Pharmaceutical and Environmental Sciences, University of Messina (Messina, Italy).

### 2.1. Ag-PMA NPs Formulation Strategy and Characterization

**Figure 1:**
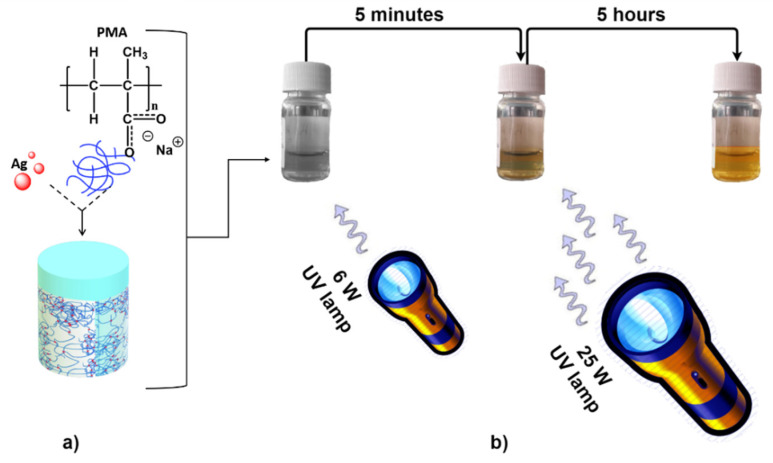
Schematic representation of the Ag-PMA colloid formulation strategy which consists in the dissolution of AgNO_3_ powder in PMA solution up to 10:1 molar ratio (**a**), followed by two-steps UV irradiation process (**b**).

A high purity (98%) silver nitrate (AgNO_3_) powder was dissolved into a 30% water solution of PMA (Poly(methacrylic acid) sodium salt, ([CH_2_C(CH_3_)(CO_2_Na)]_n_, Mw = 9500); up to 10:1 (AgNO_3_/PMA) molar ratio (Figure 1a). Ag NPs stabilized in PMA matrix were fabricated via reduction processes under a two-step UV irradiation to control the kinetics of NPs nucleation as well as to obtain a narrow size distribution [40,41]. In the first step, the mixture has been exposed for 1 h to a 6 W UV lamp irradiation (estimated density of 470 nW/cm^2^). After 5 min of exposure, the reduction in Ag^+^ ions by PMA leads to a change of the color of the solution from colorless to yellow (see Figure 1b). In a second step, the mixture has been exposed for 5 h to 25 W UV lamp irradiation (estimated density 378 mW/cm^2^). During this process, PMA aqueous solution acted as capping and reducing agent. The solution color turned to dark orange (see Figure 1b).

The stability of the obtained solution has been monitored, after one month in air and dark conditions, carrying out Dynamic Light Scattering (DLS) measurements. A Horiba NanoParticle Analyzer SZ-100(Horiba, Grenoble, France), working in the 0.3 nm–8 µm range, was used to determine Zeta potential values. The process results have been also monitored by UV–vis optical spectroscopy and Scanning Electron Microscopy working in Transmission mode (STEM) (ZEISS, Hamburg, Germany). The Perkin-Elmer Lambda 750 spectrometer (Perkin-Elmer, Waltham, MA, USA) was used to collect optical absorption spectra. Further, a Zeiss-Gemini 2 electron microscope, operating at the accelerating voltages of 150 kV and 30 kV, has allowed aquirement of SEM and STEM images, respectively. To this purpose, a drop of each suspension was deposited on a 400 mesh carbon support sputter-coated with chromium and left to dry at room temperature for 2 h.

X-ray diffraction (XRD) patterns of the sample were recorded using a Bruker AXS D8 advanced diffractometer (Bruker, Karlsruhe, Germany)with CuKα radiation (=1.5406 Angstrom) in the range of 20–80°. The thermogravimetric weight loss curve was recorded as a function of temperature using a Mettler Toledo TGA(Mettler Toledo, Greifensee, Switzerland) apparatus in air. The sample weight was 2.5 mg. The sample was heated at the maximum programmed heating rate of 10 °C min^−1^. The weight loss was calculated by the difference between the weights at room temperature (RT) and at 500 °C. The balance sensitivity was 0.1 mg.

### 2.2. Drug Loading

The drug loading process was performed as reported in Ref [50]. Briefly, 20 mL of SFT solution at the final concentration of 200 ppm were prepared. Then, 500 mg of Ag-PMA sample were added into the drug solution and dispersed by ultrasonication treatment (Sonics VCX 130). The mixture was stirred at room temperature for 24 h.

The amount of SFT loaded in Ag-PMA system was spectrophotometrically estimated by the following strategy. A weight amount (1 mg) of Ag-PMA loaded with SFT was well dissolved in 4 mL of PBS by sonication treatment and centrifuged at 6000 rpm for 45 min. The precipitate was collected, repeatedly washed to remove the free drug and finally lyophilized. The nanocomposite was dispersed in DMSO/PBS solution (1:99) and UV–vis spectra were recorded, following the drug absorbance signal at the wavelength of 265 nm.

Finally, a drug loading (DL%) of 5.5% and an encapsulation efficiency (EE%) at about 60% were respectively computed by following the equations reported below [51]:

− DL% = (drug weight encapsulated in the NPs/weight of the NPs) × 100
− EE% = (drug weight encapsulated in the NPs/weight of drug used in encapsulation strategy) × 100

### 2.3. Ag-PMA Nanofibres Preparation and Characterization

Electrospinning process was selected to prepare nanofibrous scaffold with a setup consisting of a high voltage supply, a syringe pump and a collector improving the procedure described in Ref. [50]. Ag-PMA colloidal solution, loaded with SFT, was forced through a coaxial spinneret by two 10-mL syringe pumps. Electrospinning parameters (i.e., polymer solution feed rate, collection distance and applied voltage) were varied to limit beads formation. The formation of beads was avoided applying a voltage of 35 kV DC between the spinneret and the collector plate, flushing the solution at a low flow rate (about 1 cc/h). Then, the fibers were collected onto an aluminum foil positioned at a distance of 10 cm from the needle tip. The morphology of the nanofibers was investigated by SEM, while Ag NPs distribution in PMA matrix was detected by EDX probe (SE: secondary electron imaging).

### 2.4. Drug Release

The dialysis method was adopted to perform the in vitro release experiments of Ag-PMA nanocolloids [52]. In details, 4 mg of Ag-PMA system loaded with SFT were dispersed in 4 mL of 10 mM PBS at pH 7.4 and sonicated for 1 h. The dialysis bag (MWCO = 3.5–5 kDa Spectra/Porr) was filled with the colloidal solution and submerged by 15 mL of PBS solution.

Conversely, the drug release experiments involving the Ag-PMA electrospun nanofibers, were performed by submerging the system in 10 mM PBS at pH 7.4, as reported in the literature [53]. In both cases the system was kept at 37 °C, under constant stirring. Then, 1 mL of the release medium was withdrawn, replaced with an equal volume of a fresh one, and finally analyzed by UV-Vis optical spectroscopy at fixed time intervals. The experiments were carried out in duplicates with a continuous He-Ne laser as source (λ = 632 nm, energy density = 21 mW/cm^2^).

## 3. Results

### 3.1. Ag-PMA Nanosystems Formulation and Characterization

**Figure 2:**
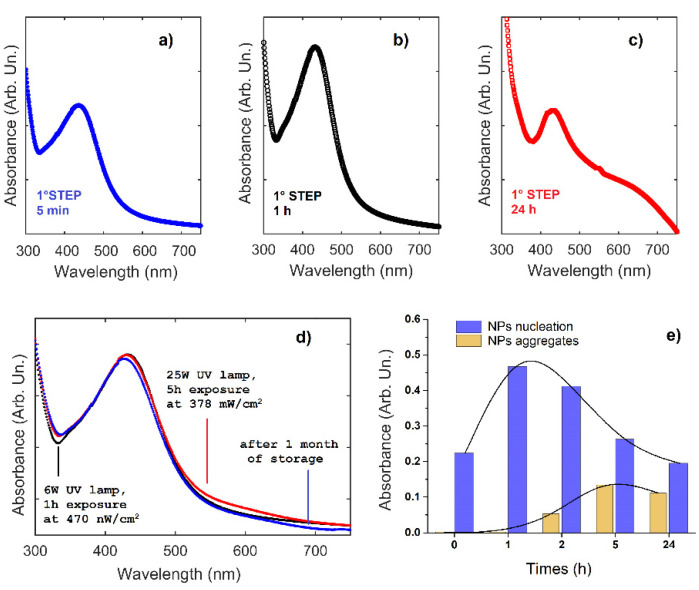
UV-vis optical absorbance spectra at different times during the first (**a**–**c**) and second (**d**) step of UV irradiation treatment to prepare Ag-PMA nanocolloid. The spectrum after one month by the second step of irradiation is also shown (**d**). The optical absorbance value vs. time is reported in panel (**e**) for the two main processes (NPs nucleation and aggregates).

A nanohybrid Ag-PMA system was prepared by a UV-vis AgNO_3_ photoreduction process [40,41], and the colloidal stability of the obtained nanocomposite was monitored at different irradiation time by carrying out UV-vis optical absorption measures. The intensity of the plasmon resonance band at about 425 nm slightly increases with irradiation time as shown in Figure 2a–c. It was also observed that, by varying the exposure time from 5 to 60 min, the peak width decreases progressively of some nanometers, while its band intensity increases (Figure 2b). At irradiation time longer than 300 min, the plasmonic peak becomes wider and another broad band around 620 nm appears (Figure 2c). These changes can be attributed to the occurrence of NPs nucleation process [54]. Assuming that the plasmonic peak is representative of the NPs nucleation, while the peak at 500 nm represents the formation of NPs aggregates, the above results suggest that the NPs aggregation process starts when the nucleation has been almost completed (see Figure 2e). It is important to remark that the plasmon intensity, which is related to the nucleation mechanism, grows up faster than the intensity of the 620 nm absorbance band. This suggests that the NP nucleation mechanism is faster than the subsequent NP growth. Considering the previous results, 1 h exposure at 470 nW/cm^2^ was chosen as the first step of the reducing process. Moreover, UV–vis optical absorbance spectra after the first and second step of irradiation are very similar (Figure 2d), indicating that the polymer reticulates during the second step strongly limit any further Ag NPs nucleation, growth, and aggregation processes.

**Figure 3:**
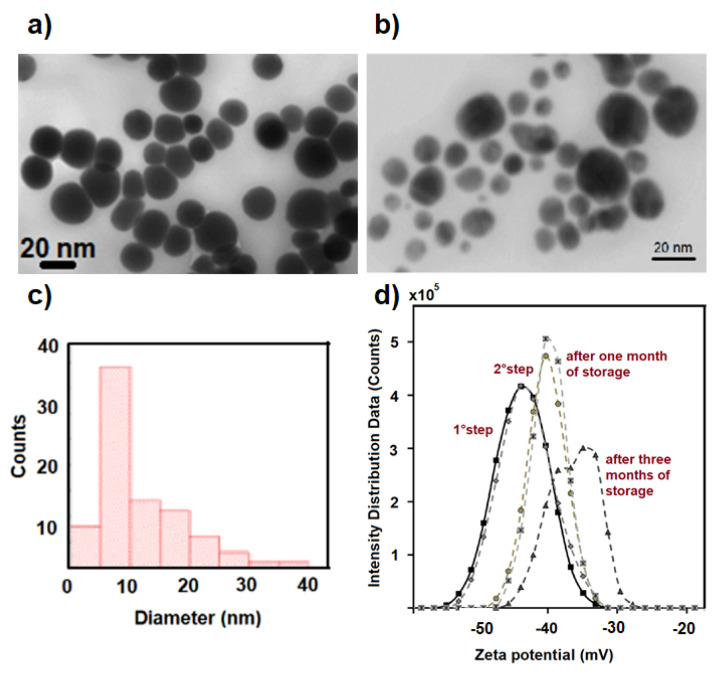
STEM images (**a**,**b**) of Ag-PMA nanocolloid (before and after one month of storage) and the corresponding size distribution histogram obtained by using Matlab Toolbox for image analysis (**c**). Zeta potential values of Ag-PMA colloidal solution at different conditions (**d**) are reported.

The stability of the obtained nanohybrid Ag-PMA colloidal system was monitored by the optical absorbance features previously described. After a one-month storage period in air and dark conditions, the size and shape of the Ag-PMA nanocolloid remain almost unchanged (see STEM images in Figure 3a–c and UV-vis optical absorption spectra in Figure 3d). The good stability of this nanohybrid system is also corroborated by the observed Zeta potential (ZP) value and the intensity distribution data. ZP value is of about −45.0 mV after the first irradiation step, about −38.0 mV after the second irradiation step and one month of storage (Figure 3d), proving the high colloidal stability of Ag-PMA system. Only after three months of storage, a bimodal intensity distribution and ZP values up to −30 mV are found.

**Figure 4:**
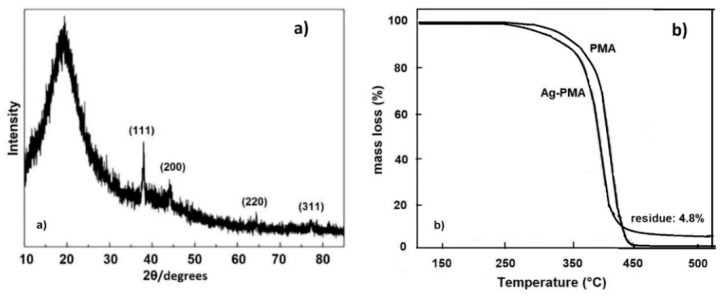
In panel (**a**) the XRD spectrum of Ag-PMA nanocolloid is reported, while in panel (**b**) the TG curves of Ag-PMA nanohybrid colloid and alone PMA, are shown.

The XRD pattern of the Ag-PMA nanocolloid was also investigated (Figure 4a). The Ag crystal phase within the PMA matrix is indicated by the XRD peaks centered at 19.35°, 37.99°, 44.06°, 64.57° and 77.29°, and corresponding to the (111), (200), (220) and (311) planes of the Ag face-centered cubic (fcc) crystal structure [55]. Furthermore, the thermal stability of Ag-PMA was evaluated by thermogravimetric analysis (TGA). TG curves showed a lower thermal stability of the hybrid Ag-PMA system respect to the pure PMA system (Figure 4b). This trend is due to the homogeneous distribution of Ag NPs in the polymer matrix (see Figure 3), which induces a rapid heating effect, aiding the decomposition of the PMA matrix [35]. A percentage residual mass of 4.8% was estimated for the Ag-PMA sample, which corresponds to the amount of Ag NPs loaded into the polymer matrix. No residual mass was detected for PMA sample.

**Figure 5:**
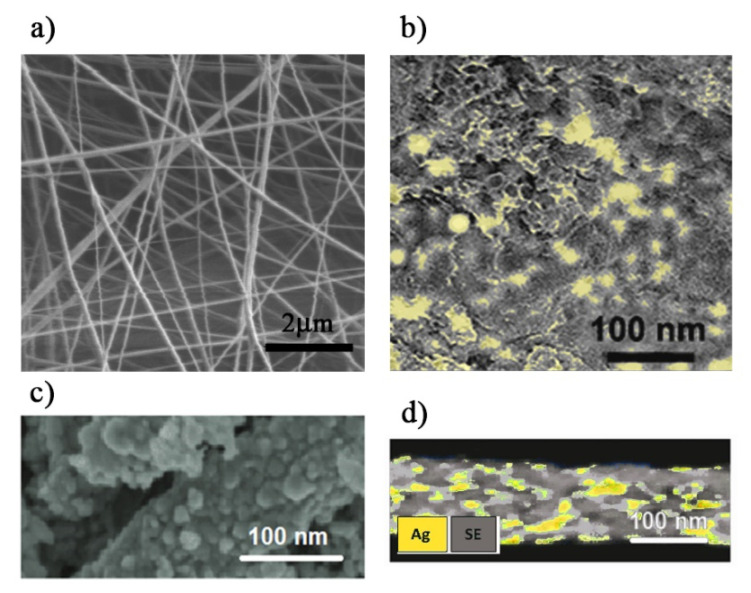
SEM images (**a**–**c**) and EDX analysis (**d**) for the Ag-PMA electrospun scaffold.

Rapid dissolution, increased drug solubility and bioavailability are important factors to control the delivery rate. One useful approach to achieve this goal is to load active pharmaceutical ingredients by the electrospinning technique, since this is a method that produces ultra-fine fibers (from micro- to nanometers of diameter), with controlled surface morphology. In our case, an electrospinning technique was used to produce the SFT-loaded Ag-PMA nanofibrous scaffold. Figure 5a shows a representative SEM image of the Ag-PMA nanofibers after an optimization of the electrospinning procedure. The nanofibers, randomly oriented, are characterized by a porous structure and a diameter of about 100–200 nm. As can be envisaged in Figure 5b, EDX probe allows visualization of a uniform distribution of Ag NPs (5–25 nm in size) along the electrospun scaffold. The pore size is less than 20 nm (Figure 5c) and Ag NPs are embedded within the porous nanostructure (Figure 5d).

### 3.2. Drug Release

To fabricate controlled drug release devices, we combined the properties of the PMA polyelectrolyte with those of Ag NPs. Ag-PMA nanocolloid was loaded with SFT, a hydrophobic drug, and a drug loading percentage (DL%) of ~5.5% and an encapsulation efficiency percentage (EE%) of ~60% were respectively evaluated [50]. Subsequently, Ag-PMA colloidal solution was electrospinned to prepare Ag-PMA nanofibers loaded with SFT. We investigated both the spontaneous drug release processes and the triggered ones. In the last cases, Ag-PMA systems were laser irradiated at 632.8 nm and displayed two different drug release profiles.

**Figure 6:**
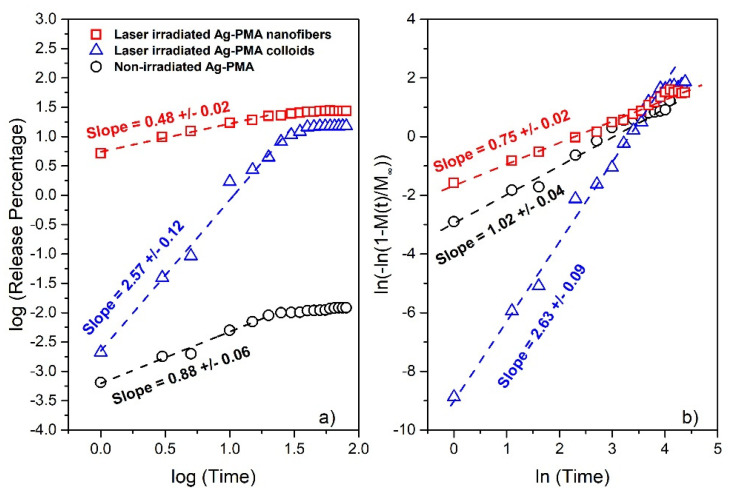
Log-log plot of the drug release percentage vs. time in hours for SFT embedded in PMA-Ag matrix at different conditions (**a**). Linear correlations, corresponding to power law behavior, can be observed only for the initial release. Weibull plot of the same data to check if the Weibull function can be used to reproduce the whole release process for all the studied conditions (**b**). Data for the non-irradiated system refer to both colloids and nanofibers. In Figure S2 of Supplementary Materials we include the comparison with the irradiated PMA samples that show no significant release performances.

To initially check for a possible simple power-law approach and infer the character of the release mechanism, we show in Figure 6a the log-log plot of the drug release percentage versus the release time for all samples. Data show a linear correlation (and then the release kinetics can be described by a power law) over a different time, up to the saturation release percentage value. The corresponding linear fits (whose slope provides the first information about the release mechanism) are also shown. We obtain a slope of 0.88 ± 0.09 for the non-irradiated system suggesting a zero order kinetic mechanism for drug release [56,57], (2.57 ± 0.12) and (0.48 ± 0.02) for the light irradiated samples (triangle symbols for the colloids and square symbols for the nanofibers system), respectively. According to theory [56], the highest estimated slope for the irradiated colloidal systems indicates that the release mechanism is quite complex since several internal collective processes may have occurred (polymer degradation, polymer swelling and so on). On the contrary, a slope of about 0.5, as that obtained for nanofibers, indicates that the initial drug release process could be described by a diffusion process [58]. Then, in Figure 6b, we report the same data in the so-called Weibull plot form (showing the linearization of the Weibull distribution reported in Equation (1) in the double logarithmic form). All that to check if the Weibull function can be efficiently used to reproduce the whole release process for all the investigated conditions [47]. The obtained slope provides a first estimation of the Weibull exponent β, better described below. As reported in Figure 6b, we obtain a slope of 1.02 ± 0.04 for the non-irradiated samples, (2.63 ± 0.09) and (0.75 ± 0.02) for the light irradiated samples (triangle symbols for the colloids and square symbols for the nanofibers system), respectively.

We used the Weibull CDF, a distribution function of wide applicability [45,59], for a statistical description of the entire release process, obtaining information on both simple and complex scenarios. Usually, simple scenarios correspond to release mechanisms based only on diffusion processes that can be described just by the Higuchi model or power law form [60]. As above mentioned, high exponents underlie the presence of complex release mechanisms such as polymer swelling plus erosion of matrix, and diffusion of drug taking place simultaneously [58,61].

The Weibull non-normalized CDF can be written as:

(1) $$M\left(t\right)=M_{\infty }\left(1-e^{-{\left(\frac{t}{\tau_{K}}\right)}^{\beta }}\right)$$

where M_∞_ corresponds to the maximum released drug amount and $\tau_{K}$ and β are constants related to the specific release mechanisms: their values have been correlated with the diffusion coefficient of matrices with high- and low-diffusivity areas [62]. The corresponding Weibull non-normalized PDF is then given by:

(2) $$f\left(t\right)=\frac{dM\left(t\right)}{dt}=M_{\infty }\frac{\beta }{\tau_{K}}{\left(\frac{t}{\tau_{K}}\right)}^{\beta -1}e^{-{\left(\frac{t}{\tau_{K}}\right)}^{\beta }}$$

The Weibull function is one of the simplest distribution models, that can be used for multi-step chain processes such as survival in cancer. In such cases, $1/\tau_{K}$ and β represent a scale factor and a shape factor, respectively [56].

When the term ${\left(\frac{t}{\tau_{K}}\right)}^{\beta }$ is small, the Weibull function can be rewritten by taking a Taylor series expansion, so obtaining:

(3) $$M\left(t\right)=M_{\infty }\left(1-e^{-{\left(\frac{t}{\tau_{K}}\right)}^{\beta }}\right)\approx M_{\infty }\left[1-\left(1-{\left(\frac{t}{\tau_{K}}\right)}^{\beta }\right)\right]=M_{\infty }{\left(\frac{t}{\tau_{K}}\right)}^{\beta }$$

that coincides with the power law form which, in turn, corresponds to the Higuchi law for β =½. Additionally, the cumulative Weibull distribution is the complementary function of the well-known stretched exponential form, widely used for the description of relaxation in disordered systems. It is called the Kohlrausch function, since it was launched by Rudolf Kohlrausch with the aim to explain the discharge of a capacitor [63], and can be generalized as:

(4) $$f\left(t\right)=e^{-{\left(\frac{t}{\tau_{K}}\right)}^{\beta }}$$

The corresponding averaged relaxation time coincides with that of the Weibull function [49] being:

(5) $$\langle \tau \rangle =\int_{0}^{\infty }\mathrm{dt}\;e^{-{\left(\frac{t}{\tau_{K}}\right)}^{\beta }}=\frac{\tau_{K}}{\beta }\Gamma \left(\frac{1}{\beta }\right)$$

where Γ is the Gamma function. The corresponding standard deviation (the square root of the variance) is given by:

(6) $$\sigma =\sqrt{\sigma^{2}}=\sqrt{\frac{\tau_{K}^{2}}{\beta }\left[2\Gamma \left(\frac{2}{\beta }\right)-\frac{1}{\beta }{\left(\Gamma \left(\frac{1}{\beta }\right)\right)}^{2}\right]}$$

## 4. Discussion

**Figure 7:**
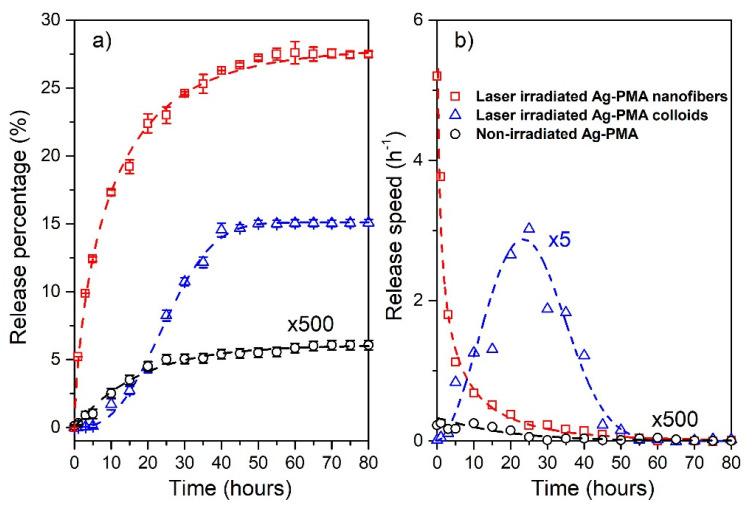
The release percentage vs. time and the corresponding best-fit performed with the use of the Weibull CDF (Equation (1)) (dashed lines) are reported in panel (**a**). The Weibull PDFs corresponding to the release data and fitted curves are reported in panel (**b**). Data for the non-irradiated system, referred to both colloids and nanofibers, are multiplied by a factor 500. The release speed data for the irradiated colloids are multiplied by a factor 5. In Figure S3 of Supplementary Materials, we include the comparison with the irradiated PMA samples that show no significant release performances.

The drug release profiles for the irradiated and non-irradiated samples and their Weibull best-fit are reported in Figure 7a. The maximum saturation values were reached after 80 h and correspond to 0.012% for the non-irradiated system, and 15.1% and 27.8% for the nanocolloids and the electrospun nanofibers, respectively. In details, 5% of the drug was released within 20 h by the nanocolloids while in just 1 h by nanofibers. Additionally, the colloids reached the maximum amount of drug released just after 40 h, whereas nanofibers continue releasing up to ~70 h. Further information was obtained from the Weibull fitting procedure setting M_∞_ = 0.012 for the non-irradiated system: 15.1 and 27.8 for external stimulated colloids and nanofibers, respectively.

For the non-irradiated release, the shape parameter β, previously described and corresponding to a first-order release [58], and the characteristic time $\tau_{K}$ were estimated to be 1.01 ± 0.07 and 17.60 ± 0.07 h, respectively. For first order kinetics, the rate of release is governed by the concentration gradient in the dissolution medium [64] and, in such cases, the mean time 〈τ〉 coincides with $\tau_{K}$. On the other side, analyzing the trend of the laser-induced drug release, we obtain:

- for the colloidal systems
  β = (2.67 ± 0.09), $\tau_{K}$ = (27.90 ± 0.04) h, 〈τ〉 = 24.8 h and σ= 8.8 h.
- for the scaffold systems
  β = (0.71 ± 0.02), $\tau_{K}$= (10.51 ± 0.03) h, 〈τ〉 = 13.1 h, and σ = 23.6 h.

A value of β ≤ 0.75 is associated with the Fick diffusion in either fractal or Euclidian spaces, while a combined mechanism (Fick diffusion and swelling controlled transport) is associated with β values in the range 0.75 < β < 1. For the shape factor β > 1, the release mechanism is quite complex: the rate of release initially increases non-linearly up to the inflection point and thereafter decreases asymptotically [58,65], as shown for the colloidal system (blue triangles and dashed line in Figure 7b corresponding to the Weibull PDF for the experimental and modeled data, respectively). For what concerns the nanofiber system, a pronounced exponential character (for which the expected high standard deviation value) is observed by the release speed (Figure 7b), indicating a quicker and a more efficient release process whose character is mainly diffusive.

As mentioned in the Introduction section, the aim of this work is to study the influence of PMA properties, Ag NPs distribution and devices morphologies on the drug release efficiency. From a future perspective, the antioxidant and/or antimicrobial properties of these systems could be tested to reach responsive packaging systems. Weibull fitting represents a best practice to reproduce the whole life-time drug release process. Moreover, it is helpful in the comparison between the release profiles of matrix systems characterized by a different morphology (core/shell nanostructures, electrospun scaffolds, etc.). However, this model has no fundamental kinetic basis and cannot elucidate the release kinetic properties adequately since there is not a single parameter that is proportional to the intrinsic dissolution rate of the releasing drug. Despite that, a subsequent correlation between the profile-defining parameters and the samples’ morphological properties (which, as is well-known, influence the dissolution time profile [66]) has been made, allowing determination of the dissolution efficiency of our nanosystems. The dissolution efficiency (DE) is defined by the Food and Drug Administration (FDA) and the European Medicines Agency (EMA) as the ratio between the area under the release profile up to a certain time (*t_f_*) and the area of the rectangle described by 100% release at the same *t_f_* [67]:

(7) $$\mathrm{DE}\;\left(\%\right)=\frac{\int_{0}^{t_{f}}M\left(t\right)\;\mathrm{dt}}{100\times t_{f}}\times 100=\frac{\int_{0}^{t_{f}\;\;\;}M_{\infty }\left(1-e^{-{\left(\frac{t}{\tau_{K}}\right)}^{\beta }}\right)\;\mathrm{dt}}{\;t_{f}}$$

where M(t) is the Weibull non-normalized CDF reported in Equation (1). Herein, we have estimated DE values (see Table 1) both at fixed times (40 and 80 h) and drug release percentages (5 and 10%), to which different release mechanisms are associated. So, an attempt at prediction dissolution for a future optimized PMA based product has been proven trying to understand dissolution mechanism, particularly determining which individual rate processes (e.g., surface dissolution, diffusive mass transfer, scaffold disintegration) are rate-limiting for the overall dissolution for the two typologies of investigated samples (colloids and nanofibers scaffolds).

As is well-known, the localized and intensive heating of Ag NPs, induced by the external laser light irradiation, results in the thermal expansion of the polymer, causing the drug diffusion [36]. This is the main mechanism reported to explain the drug release behavior in polymeric matrix decorated with metal NPs. In our case, the laser light is far from Ag SPR, and the drug release efficiency coming from the electrospun nanofibers is strongly increased with respect to the colloids and to the non-irradiated samples. The temperature increasing during the laser irradiation causes a rearrangement of non-covalent interactions in the PMA matrix leading to the hydrogen bonding dissociation of PMA complex units. In this transition phase, the hydrophilic and hydrophobic interactions within the polymer matrix, as well as between the polymer sidechains and the surrounding water molecules play a key role [68]. In fact, hydrophobic segments are surrounded by cage-like structures formed by water molecules bridged by hydrogen bonds. Upon increasing the temperature, thermal fluctuations destabilize the cage-like structures, inducing first the aggregation into hydrophobic clusters (known as volume phase transition) from which water molecules are expelled, and then the breakdown of hydrophobic segments for their direct contact with water molecules. All these processes are favored in the electrospun nanofibers where the increased surface area improved the sample wettability; in turn, the formation of hydrophobic barriers upon contact with water was avoided, leading to an enhancement of bioavailability of poor water-soluble drugs [18]. Hence, the porous nanofibers morphological reorganization (i.e., the increased surface/volume ratio) has resulted in significant changes of PMA surface properties, thus, creating a switchable polymeric system containing also an active element, responsive to the laser light external stimulus. From here, the release of SFT is faster in the electrospun sample than that of the colloid due to a higher diffusion rate.

In perspective, taking into account these preliminary results, a link between dissolution and clinical performance would ideally be established also providing a potential manufacturing tool for early-stage formulation development, a robust control strategy, real-time release testing, and flexibility toward post-approval changes. Furthermore, this work is interesting for researchers engaged in the fabrication of antimicrobial metallic-polymer-based nanocomposite system. The promising release data found about Ag-PMA systems led us to conduct antibacterial tests on *Escherichia coli* and *Staphylococcus aureus* to qualitatively evaluate their antibacterial activity. These preliminary results, shown in the Supplementary Materials (see Figure S1), are pivotal in view of the mentioned possible technological applications of these systems. In addition, despite the interesting antibacterial activity, tests on cytotoxicity, necrosis, and apoptosis using cell lines isolated from various human and animal tissues will be carried out.

## 5. Conclusions

Herein, Ag-PMA nanofiber scaffold was prepared by a safe and eco-friendly electrospinning procedure, starting from Ag-PMA colloidal solutions. Ag-PMA electrospun nanofibers were tested to be used as a carrier for a laser light-controlled drug release compared with the same colloidal system. The release profiles have been analyzed in terms of the Weibull statistical distribution function, which allows achievement of information about the processes that, damaging the stability of PMA compact system, govern the release mechanism. We believe that the reported results are interesting in perspective to establish a link between dissolution and clinical performance and are also considered important, from a manufacturing point of view, to fabricate controlled release packaging, i.e., a new generation of packaging materials that can release active compounds, such as antimicrobials and antioxidants, at desirable rates to extend the shelf life of a wide variety of foods. However, further studies closely combining the features of Ag-PMA found here with the potential applications related to technological/biomedical demands are still necessary and already scheduled.

## Figures and Tables

**Table 1:** DE values estimated both at fixed times (40 and 80 h) and drug release percentages (5 and 10%), to which different release mechanisms are associated.

	Time (hours)	Release (%)	DE (%)
Nanofibers	1	5	3
	3.5	10	6.3
	40	26	20.2
	80	28	23.6
Colloids	20	5	1.5
	29	10	3.4
	40	14	5.9
	80	15	10.4

## Data Availability

The data presented in this study are available on request from the corresponding author.

## Supplementary Materials

The following are available online at https://www.mdpi.com/article/10.3390/polym13172897/s1, Figure S1. Photos of bacterial culture plates of *E. coli* and *S. aureus* under PMA and Ag-PMA are shown in comparison with the control and Ag NPs; Figure S2. Log-log plot of the drug release percentage vs. time in hours for SFT embedded in PMA-Ag matrix at different conditions including irradiated PMA samples (a). Weibull plot of the same data (b). Data for the non-irradiated system refer to both colloids and nanofibers; Figure S3. The release percentage vs. time and the corresponding best-fit performed with the use of the Weibull CDF (Equation (1) of the paper) (dashed lines) are reported in panel a. The Weibull PDFs, describing the release speed profile corresponding to the release data and fitted curves, are reported in panel b. Data for the non-irradiated system, corresponding to both colloids and nanofibers, are multiplied by a factor 500. Release data of PMA samples are multiplied by a factor 50 and the corresponding release speed by a factor 250. Finally, the release speed data for the irradiated colloids are multiplied by a factor 5.
