# Supplementary material for: Weibull Modeling of Controlled Drug Release from Ag-PMA Nanosystems
Source: Polymers (Basel). 2021 Aug 27;13(17):2897. doi: 10.3390/polym13172897 (PMC8434431; doi:10.3390/polym13172897)
Supplement: Supplementary file 1 [file polymers-13-02897-s001.zip › polymers-1343454-SI.pdf]

Supplementary Materials: Weibull modeling of controlled drug release from Ag-PMA nanosystems

Carmelo Corsaro , Giulia Neri , Angela Maria Mezzasalma and Enza Fazio *

1. Antibacterial tests

Here we report preliminary antibacterial tests carried out to qualitative evaluate antibacterial activity Ag-PMA tested against *Escherichia coli* (*E. coli*) and *Staphylococcus aureus* (*S. aureus*).

Photos of bacterial culture plates of *E. coli* and *S. aureus* under PMA and Ag-PMA are shown in Fig. S1. In comparison with the control, dense colonies of both bacteria have been observed on the plates in which PMA was used as antibacterials agent. Otherwise, a very low number of bacteria can be counted on the plates in which bacteria are exposed to Ag-PMA nanocolloids. Thus, no significant antibacterial activity occurs in PMA, while it is relevant in the hybrid Ag-PMA nanocolloids. In order to test the action of PMA to improve Ag NPs antibacterial activity, we have prepared, by the pulsed laser ablation procedure, a water colloidal solution of Ag NPs following a procedure analogous to that reported in Ref. [1]. The silver nitrate target used in the ablation process was obtained pressing the silver nitrate powder into a disk at room temperature. The number of bacteria is reduced when exposed only to the Ag NPs but, in any case, it is not as in the case of the Ag-PMA system. Therefore, the remarkable antibacterial activity could be explained considering the improved Ag-PMA stability, since the carboxyl group (-COOH) in polymer chains could have arranged Ag NPs, with a consequent delaying effect on the release of Ag^{+} ions and, in turn, a reduced oxidative of Ag NPs.

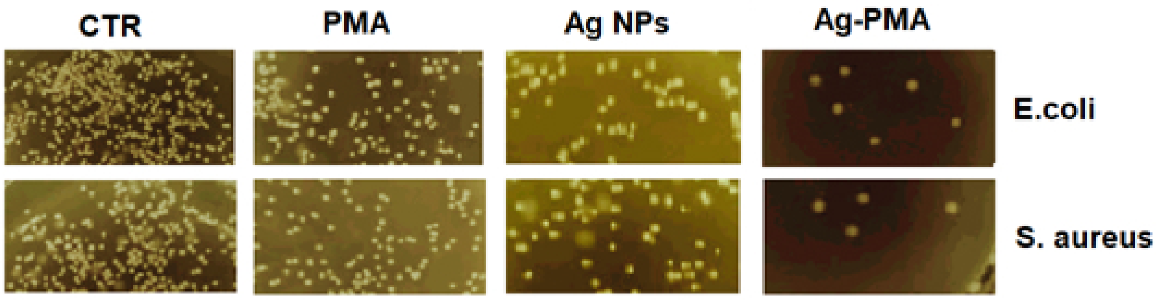

Figure S1. Photos of bacterial culture plates of *E. coli* and *S. aureus* under PMA and Ag-PMA are shown in comparison with the control and Ag NPs.

2. Drug release comparison with respect to PMA samples

Here we report the comparison of the drug release performances of the Ag-PMA samples with that of PMA. First we plot in Fig. S2 an initial check of the release behaviour in terms of the power-law and Weibull plot. In fact, Fig. S2a shows the log-log plot of the drug release percentage vs time in hours for SFT embedded in PMA-Ag matrix at different conditions compared with that of irradiated PMA samples. Linear correlations, corresponding to power law behavior, can be observed only for the initial release. Similarly, Fig. S2b shows the Weibull plot of the same data to check if the Weibull function could be used to study the whole release process for all the studied conditions. Note that data for the non-irradiated system refer to both colloids and nanofibers.

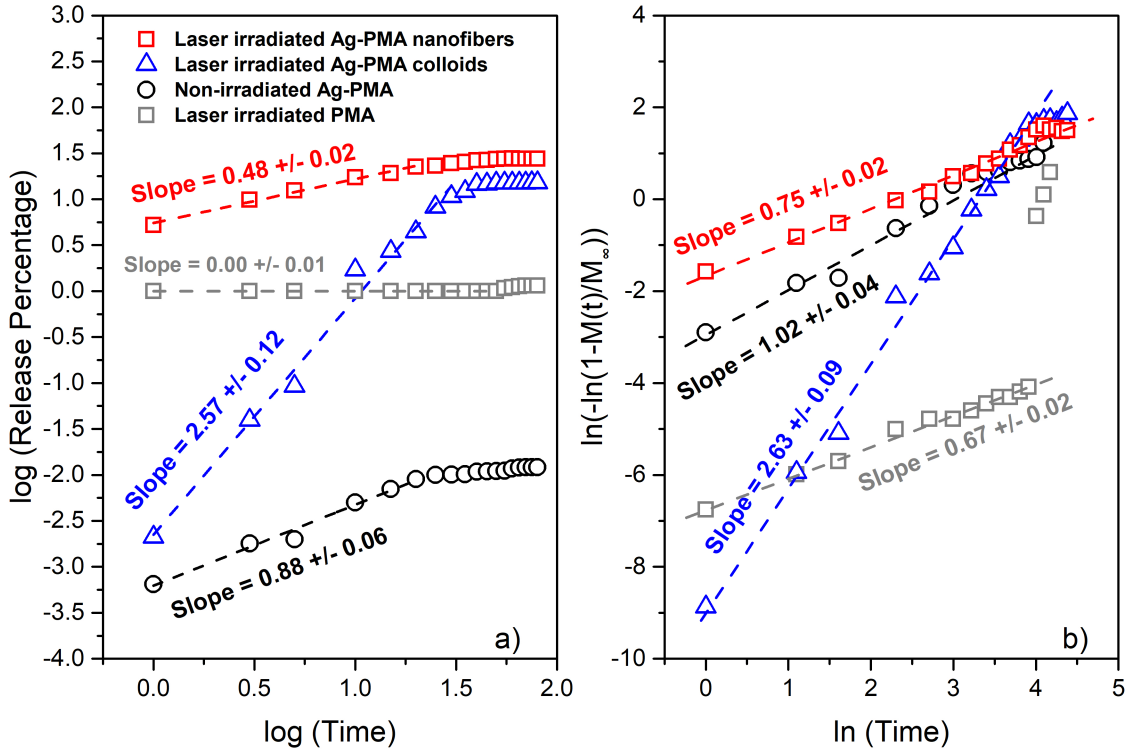

Figure S2. Log-log plot of the drug release percentage vs time in hours for SFT embedded in PMA-Ag matrix at different conditions including irradiated PMA samples (a). Weibull plot of the same data (b). Data for the non-irradiated system refer to both colloids and nanofibers.

Then we plot in Fig. S3 the Weibull fitting of the release process (panel a) and the corresponding release speed profile (panel b) including data from irradiated PMA samples. We recall that the release process can be statistically interpreted as a Cumulative Distribution Function (CDF) whose derivative corresponds to the Probability Distribution Function (PDF) of the same process.

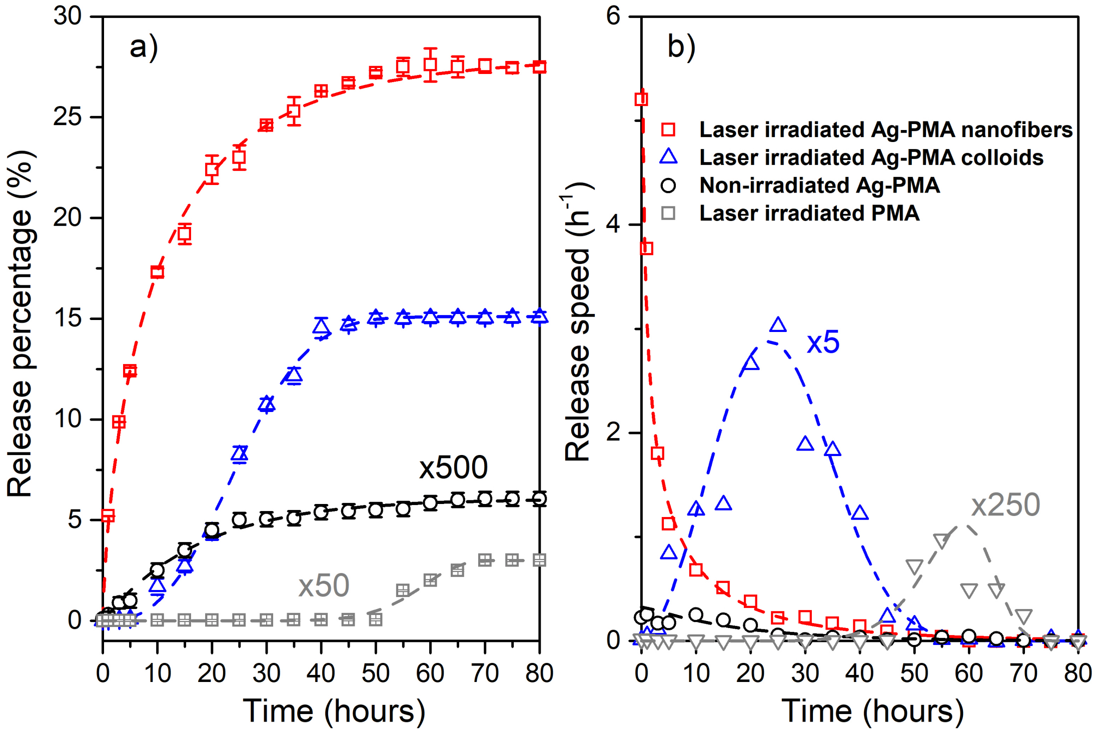

Figure S3. The release percentage vs time and the corresponding best-fit performed with the use of the Weibull CDF (eq. 1 of the paper) (dashed lines) are reported in panel a. The Weibull PDFs, describing the release speed profile corresponding to the release data and fitted curves, are reported in panel b. Data for the non-irradiated system, corresponding to both colloids and nanofibers, are multiplied by a factor 500. Release data of PMA samples are multiplied by a factor 50 and the corresponding release speed by a factor 250. Finally, the release speed data for the irradiated colloids are multiplied by a factor 5.

As reported in the main text, the irradiated PMA samples do not show significant release performances. In fact, as it shown in Fig S3, only after about 50 hours of irradiation, the heat so induced is able to break the weakest bonds within the polymer chains allowing only a very small release of the SFT (less than 0.1%).

Abbreviations

The following abbreviations are used in this Supplemental:

NPs	NanoParticles
PMA	poly-methacrylic acid,5 sodium salt
Ag	Silver
SFT	Sorafenib-Tosylate
CDF	Cumulative Distribution Function
PDF	Probability Distribution Function

References

1. Fazio, E.; Mezzasalma, A.; D'urso, L.; Spadaro, S.; Barreca, F.; Gallo, G.; Neri, F.; Compagnini, G. N-TiO_{2-x} Nanocatalysts: PLAL Synthesis and Photocatalytic Activity. *Journal of Nanomaterials* **2020**, *2020*, 1–10. doi:10.1155/2020/2901516.
